# Failure of annexin-based apoptosis imaging in the assessment of antiangiogenic therapy effects

**DOI:** 10.1186/2191-219X-1-26

**Published:** 2011-11-17

**Authors:** Wiltrud Lederle, Susanne Arns, Anne Rix, Felix Gremse, Dennis Doleschel, Jörn Schmaljohann, Felix M Mottaghy, Fabian Kiessling, Moritz Palmowski

**Affiliations:** 1 Experimental Molecular Imaging, Medical Faculty, RWTH Aachen University, Pauwelsstraße 20, Aachen, 52074, Germany; 2 Department of Nuclear Medicine, Medical Faculty, RWTH Aachen University, Pauwelsstraße 30, Aachen, 52074, Germany; 3 Department of Nuclear Medicine, Maastricht University Medical Center, P.O. Box 616, Maastricht, 6200 MD, The Netherlands; 4 Department of Radiology, Medical Faculty, RWTH Aachen University, Pauwelsstraße 30, Aachen, 52074, Germany

**Keywords:** angiogenesis, apoptosis, optical imaging, therapy monitoring, ultrasound

## Abstract

**Background:**

Molecular apoptosis imaging is frequently discussed to be useful for monitoring cancer therapy. We demonstrate that the sole assessment of therapy effects by apoptosis imaging can be misleading, depending on the therapy effect on the tumor vasculature.

**Methods:**

Apoptosis was investigated by determining the uptake of Annexin Vivo by optical imaging (study part I) and of ^99 m^Tc-6-hydrazinonicotinic [HYNIC]-radiolabeled Annexin V by gamma counting (study part II) in subcutaneous epidermoid carcinoma xenografts (A431) in nude mice after antiangiogenic treatment (SU11248). Optical imaging was performed by optical tomography (3D) and 2D reflectance imaging (control, *n *= 7; therapy, *n *= 6). Accumulation of the radioactive tracer was determined *ex vivo *(control, *n *= 5; therapy, *n *= 6). Tumor vascularization was investigated with an optical blood pool marker (study part I) and contrast-enhanced ultrasound (both studies). Data were validated by immunohistology.

**Results:**

A significantly higher apoptosis rate was detected in treated tumors by immunohistological terminal deoxynucleotidyl transferase-mediated dUTP nick end labeling staining (area fraction: control, 0.023 ± 0.015%; therapy, 0.387 ± 0.105%; *P *< 0.001). However, both 2D reflectance imaging using Annexin Vivo (control, 13 ± 15 FI/cm^2^; therapy, 11 ± 7 FI/cm^2^) and gamma counting using ^99 m^Tc-HYNIC-Annexin V (tumor-to-muscle ratio control, 5.66 ± 1.46; therapy, 6.09 ± 1.40) failed in showing higher accumulation in treated tumors. Optical tomography even indicated higher probe accumulation in controls (control, 81.3 ± 73.7 pmol/cm^3^; therapy, 27.5 ± 34.7 pmol/cm^3^). Vascularization was strongly reduced after therapy, demonstrated by contrast-enhanced ultrasound, optical imaging, and immunohistology.

**Conclusions:**

The failure of annexin-based apoptosis assessment *in vivo *can be explained by the significant breakdown of the vasculature after therapy, resulting in reduced probe/tracer delivery. This favors annexin-based apoptosis imaging only in therapies that do not severely interfere with the vasculature.

## Background

Apoptosis has important functions for tissue homeostasis and is dysregulated in a variety of diseases [1,2]. Whereas apoptosis is increased in cardiovascular and neurodegenerative disorders, insufficient apoptosis occurs in autoimmune diseases, and the pronounced loss of apoptosis is a hallmark of cancer tissue [1,2]. On the other hand, efficient cancer therapies like chemotherapy, radiation, or antiangiogenic treatment induce apoptosis in the tumor tissue. Thus, the detection of apoptosis, especially by noninvasive imaging technologies, is potentially of great interest for disease diagnosis, monitoring of the disease course, as well as treatment response. One characteristic event in apoptosis is the externalization of phosphatidylserines [PS] at the plasma membrane. The binding of the multifunction protein Annexin V to PS has been used for diagnostic purposes [2]. Several Annexin V-based imaging probes or tracers have been developed for the *in vivo *detection of apoptosis by radionuclide, optical, and magnetic resonance [MR] imaging techniques [1,3-9].

Most clinical experience has been gained with the radioactive tracers ^99 m^Tc-Annexin V [10,11] and ^99 m^Tc-6-hydrazinonicotinic [HYNIC]-radiolabeled Annexin V, the latter being used in phase II/III trials for determining the efficacy of chemotherapy in cancer patients [1]. In animal studies, Cy5.5-labeled Annexin V is often used for optical apoptosis imaging, including the monitoring of antitumorigenic therapies [12-14]. Although annexin-based apoptosis imaging has been applied for assessing the effects of chemo- or radiotherapy, it has not been applied to the best of our knowledge for monitoring antiangiogenic therapy effects. Therefore, we investigated near infrared [NIR]-apoptosis imaging and the uptake of ^99 m^Tc-HYNIC-Annexin V for assessing antiangiogenic therapy effects in subcutaneous A431 xenografts. SU11248 was used as antiangiogenic drug, a multi-targeted receptor tyrosine kinase inhibitor that blocks the vascular endothelial growth factor receptors, the platelet-derived growth factor receptors, and additional receptor tyrsosine kinases. A431 is a squamous cell carcinoma model [SCC] which responds very sensitively towards SU11248 [15,16]. We demonstrate that the *in vivo *assessment of therapy response in cancer by apoptosis imaging can be misleading, depending on the effect of the therapy on tumor vascularization.

## Methods

### Tumor inoculation and antiangiogenic therapy

Human epidermoid carcinoma xenografts were induced by a subscutaneous injection of 4 × 10^6 ^A431 cells (ATTC) in the right hind limb of female nude mice as described [15,17]. After 10 days of tumor growth, the animals were divided randomly into a control group (*n *= 7 for study I; *n *= 5 for study II) and a therapy group (*n *= 6 for study I; *n *= 6 for study II). Antiangiogenic treatment was performed by daily i.p. injection of 50 mg/kg body weight of SU11248 (Pfizer, Inc., New York City, NY, USA; dissolved in 60 μl DMSO and 30 μl PBS) for 4 days. Untreated animals were used as controls since application of equimolar concentrations of the respective solvents had no effects on tumor growth as described [15].

### Study design and imaging protocols

In a first study, we assessed apoptosis *in vivo *using the near infrared fluorescence [NIRF] probe Annexin Vivo. In parallel, the tumor vascularization was analyzed by NIRF imaging using AngioSense as blood pool contrast agent and by contrast-enhanced ultrasound.

In a second study, apoptosis was investigated by *ex vivo *gamma counting of tumors using the radioactive tracer ^99 m^Tc-HYNIC-Annexin V. In parallel, tumor vascularization was assessed *in vivo *using contrast-enhanced ultrasound. Imaging and radioactive measurements were performed at day 4 of therapy. This treatment period results in a significant breakdown of tumor vessels in A431 tumors as previously observed [18,19]. The following examination protocols were applied.

#### Ultrasound

The tumor volume was determined by non-contrast-enhanced ultrasound with a small animal ultrasound system at 25 MHz (Vevo770, RMV710B-transducer; VisualSonics, Toronto, Canada) by surface rendering.

For assessing the vascularization, contrast-enhanced ultrasound was performed at the above mentioned scanning system as described [18]. Animals were anesthetized with 1.5% isoflurane. A concentration of 1 × 10^8 ^of self-made polybutylcyanoacrylate microbubbles in 50 μl were intravenously injected as described [18]. Imaging (10 frames per second, 16% power) was performed during the injection of the microbubbles, and cine loops of approximately 20 s length were stored for consecutive analysis. Post-processing of the cine loops was performed by the 2D maximal-intensity-over-time technique as described [18].

#### Fluorescence molecular tomography

Fluorescence molecular tomography [FMT] was performed using a wave-type specific scanner for transillumination, reflectance, and absorption as described [20] (FMT 2500; PerkinElmer Inc., Waltham, MA, USA). Each mouse received intravenously 2 nmol of Annexin Vivo 750 (PerkinElmer Inc., Waltham, MA, USA) 2 h prior to imaging and 2 nmol of AngioSense 680 (PerkinElmer Inc., Waltham, MA, USA) immediately before imaging. Annexin Vivo 750 is a NIR probe that selectively binds phosphatidylserine exposed in the outer leaflet of the cell membrane during the early stages of apoptosis. AngioSense 680 is a NIRF agent that remains localized in the vasculature for 0 to 4 h. In order to guaranty the assessment of an intravascular probe, FMT measurements were performed directly after the injection of AngioSense. This allowed a better comparison with contrast-enhanced ultrasound where microbubbles serve as a purely intravascular contrast agent (due to their size). The mice were anesthetized with isoflurane during imaging and fixed at a definite position in a two-modality animal bed (CT Imaging GmbH, Erlangen, Germany). Quantitative 3D tomography data (in picomoles) as well as fluorescence intensities [FI] of 2D reflectance images were acquired. For 2D reflectance data, the FI in a similar region of interest [ROI] in the hind limb muscle on the contralateral side was subtracted from the FI of the tumor in order to reduce the background signal.

#### Computed tomography

For aiding in tumor localization and ROI placement, a micro computed tomography [CT] scan was performed directly after the FMT measurement using the dual energy system Tomoscope Duo CT (CT Imaging GmbH, Erlangen, Germany). The mice were kept anesthetized and fixed in the animal bed as for FMT. The following scan protocol was used: Both tubes ran at energies of 65 kV. Each flat panel detector acquired 720 projections at 25 frames per second. A full rotation was performed with a total scan time of 29 s. A Feldkamp algorithm was used for image reconstruction with an isotropic voxel size of 70 μm and a sharp reconstruction kernel (T60). After a total time of a maximum of 10 min for the FMT and CT measurements, the mice were sacrificed and the tumors were resected.

#### Quantitative uptake of ^99 m^Tc-HYNIC-Annexin V in tumors and muscle

For assessing apoptosis by a radioactive probe, we employed the tracer ^99 m^Tc-HYNIC-Annexin V as described [3]. The preparation of ^99 m^Tc-HYNIC-Annexin V was adapted to that of Blankenberg et al. [3]. In brief, 200 μl of ^99 m^TcO4 was mixed with 150 μl of HYNIC-Annexin V-tricine solution (41 μg HYNIC-Annexin V, 115 mM tricine). After dilution with 130 μl saline, 20 μl stannous chloride (1 mg/ml in 0.05 M HCl) was added, and the reaction mixture was incubated for 15 min at room temperature. The radiochemical purity was determined by instant thin layer chromatography.

Ten to fifteen megabecquerel of ^99 m^Tc-HYNIC-Annexin V (0.0575 nmol) was injected intravenously into each mouse 2 h prior to sacrifice. For quantitative determination of the tracer uptake, tumor, liver, and muscle tissues were resected and measured *ex vivo *by gamma counting (Wizard^2^; PerkinElmer Inc., Waltham, MA, USA). The number of counts measured in the tumor, liver, and muscle tissues was corrected for the injected dose per animal and the radioactive decay (*t*_1/2 _(^99 m^Tc) = 6.01 h). The tracer uptake was expressed as tumor-to-muscle ratio normalized to tissue weight.

#### Indirect immunofluorescence

For validation, resected tumors were frozen in liquid nitrogen vapor and cut in 8- to 10-μm slices. Fixation of the frozen sections and the staining procedure were performed as described [21]. Primary antibodies against CD31 (rat anti-mouse PECAM-1; BD Biosciences, Heidelberg, Germany) for endothelial cell staining, alpha smooth muscle actin [SMA] (biotinylated mouse anti-SMA; Progen Biotechnik GmbH, Heidelberg, Germany) for staining of mature vessels, and collagen IV (rabbit anti-collagen IV; Novotec, Saint Martin La Garenne, France) for vessel staining, as well as corresponding secondary antibodies were used [21]. Cell nuclei were counterstained by 4',6-diamidino-2-phenylindole [DAPI] (Invitrogen, Carlsbad, CA, USA). Apoptotic cells in the tissue were detected by terminal deoxynucleotidyl transferase-mediated dUTP nick end labeling [TUNEL] staining using the '*In Situ *Cell Death Detection Kit, TMR red' (Roche Diagnostics GmbH, Mannheim, Germany). Stained sections were examined and photographed with the Zeiss Axio Imager M2 (Carl Zeiss MicroImaging GmbH, Köln, Germany).

#### Morphometric analysis

Blood vessel density in s.c. tumors was quantified by determining the ratio of the CD31-stained area to the total tumor area within the respective field of view. Four sections at ×10 magnification were analyzed for covering the whole tumor area. The apoptotic cell rate was determined by dividing the TUNEL-positive area by the total DAPI-stained area of the tumor region (stated in percent).

### Statistics

A two-tailed Student's *t *test was applied for data analysis using GraphPadPrism 5.0 (Graph-Pad, San Diego, CA, USA). *P *< 0.05 was considered as statistically significant (*) and *P *< 0.001 as highly significant (**).

## Results

### *In vivo *optical apoptosis imaging after antiangiogenic therapy

**Figure 1:**
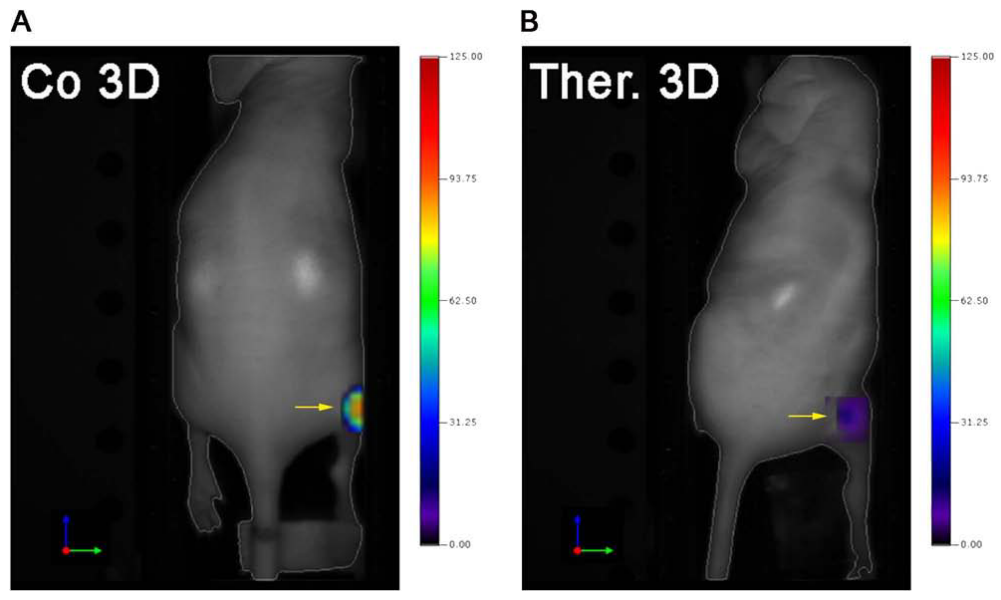
**Lower Annexin Vivo accumulation in SU11248-treated SCC xenografts**. 3D-FMT images showing the Annexin Vivo concentrations in A431 tumors of representative (**A**) control and (**B**) therapy animals. Tumors are marked with arrows; the color-bar represents the concentrations of the probe in nM.

**Figure 2:**
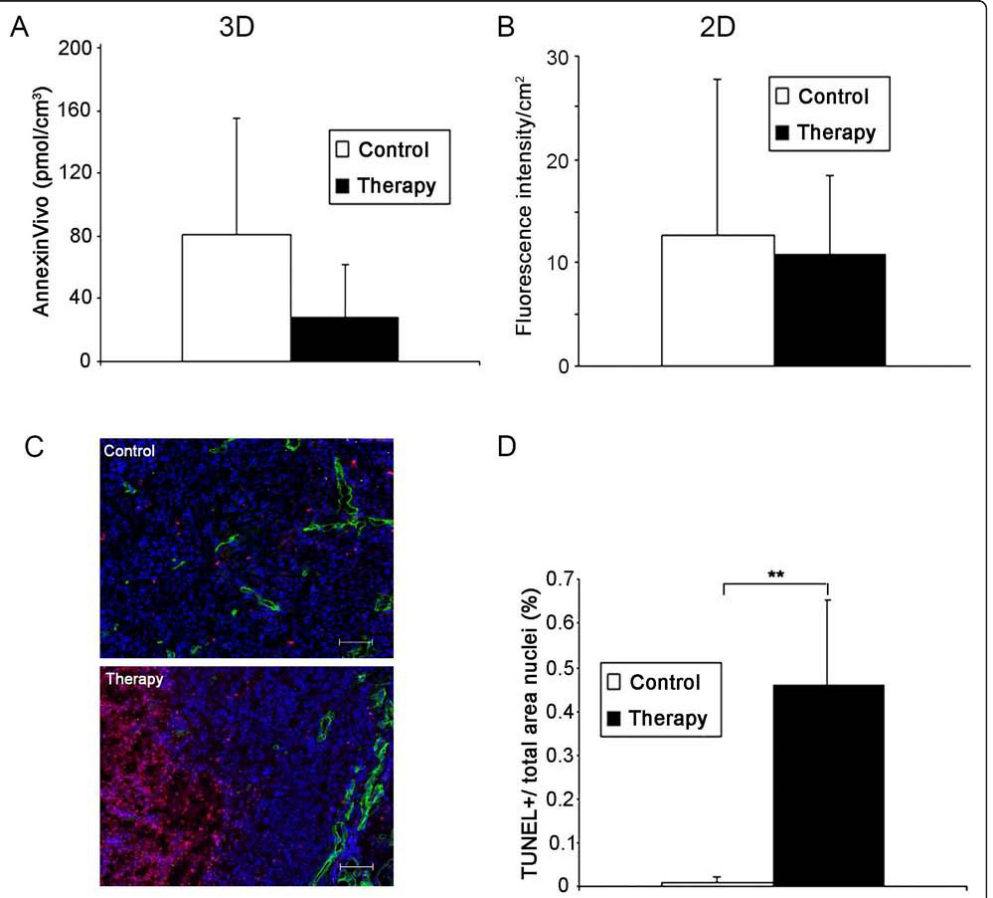
**Low Annexin Vivo accumulation contradicts the high apoptotic rate in corresponding tumor sections**. (**A**) 3D data analysis reveals a higher mean concentration of Annexin Vivo in the untreated control tumors (control, *n *= 7; therapy, *n *= 6), *P = *0.13. (**B**) 2D reflection data confirm slightly higher mean signal intensity in the control group (same animal number per group), *P = *0.79. (**C**) TUNEL staining of corresponding tumor sections demonstrates a higher number of apoptotic cells in the SU11248-treated tumors, TUNEL-positive cells in red, counterstaining of vessels by collagen IV (green), and nuclei stained by DAPI (blue), bar = 100 μm; (**D**) Quantification confirms a significantly higher ratio of the TUNEL-positive area fraction in treated tumors, **P < 0.001.

Since the benefits of cancer therapies can be attributed to the induction of apoptosis and necrosis in the tumor tissue [1], noninvasive apoptosis imaging is of great clinical interest for controlling therapy effects. Therefore, we analyzed in a first study the accuracy of apoptosis imaging by FMT using Annexin Vivo after a short-term treatment of SCC xenografts with SU11248 (4 days). At this early stage of therapy, the tumor volume was only slightly smaller in treated tumors compared with untreated controls (therapy, 28.4 mm^3 ^± 10.6; control, 36.5 mm^3 ^± 15.2), as determined by US. Surprisingly, reconstructed 3D images showed a stronger signal of Annexin Vivo in control tumors than in treated ones (Figure 1A, B). Quantification of the 3D data (control group, *n *= 7; therapy group, *n *= 6; Figure 2A) revealed 81.3 ± 73.7 pmol Annexin Vivo/cm^3 ^in control tumors and 27.5 ± 34.7 pmol/cm^3 ^in treated ones (*P *= 0.13). The average 2D FI per area was also slightly higher in tumors of the control group (Figure 2B; control, 13 ± 15 FI/cm2; therapy, 11 ± 7 FI/cm^2^; *P *= 0.79). The *in vivo *data were in clear contrast to immunohistology. A strongly enhanced number of TUNEL-positive apoptotic cells were detected on sections of treated tumors. Apoptotic cells were predominantly located in the tumor center, where only very few vessels were remaining (Figure 2C). Quantification of the TUNEL-positive area fraction demonstrated a significantly higher apoptotic cell rate in the treated group (Figure 2D; TUNEL-positive area: control, 0.011 ± 0.014%; therapy, 0.461 ± 0.194%; **P < 0.001).

### Assessment of tumor vascularization after antiangiogenic therapy

The low accumulation of Annexin Vivo in the treated tumors despite their high apoptotic rate suggested either problems in biodistribution or limitations in probe delivery to the target tissue. Since the probe is distributed via the blood circulation and antiangiogenic therapy affects the tumor vasculature, we assessed the degree of vascularization in treated and untreated tumors. Tumor vascularization was analyzed *in vivo *with FMT using AngioSense as a blood pool marker and with contrast-enhanced ultrasound.

**Figure 3:**
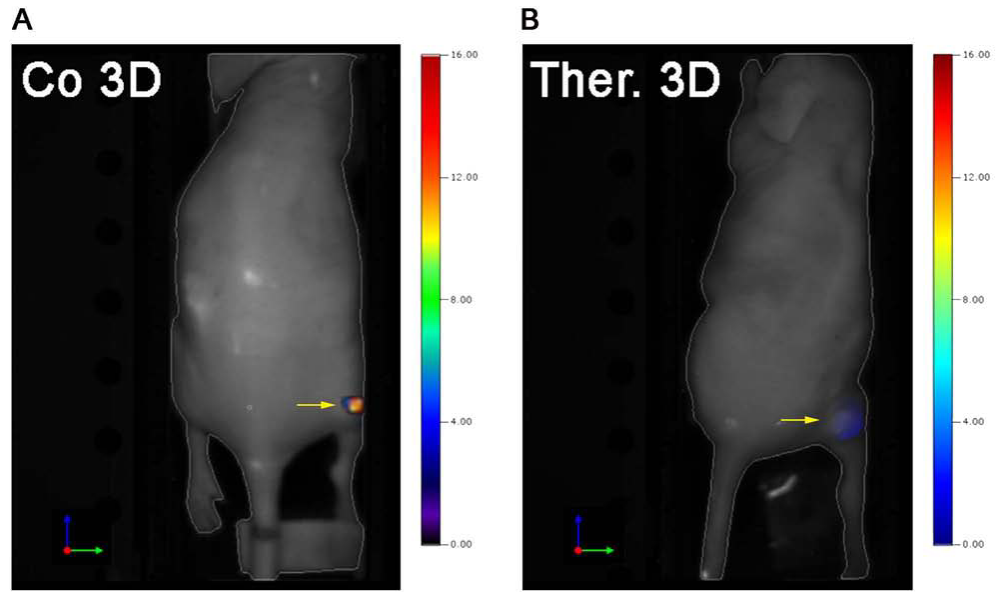
**Higher accumulation of AngioSense in untreated control tumors**. 3D-FMT images showing the AngioSense concentrations in tumors of representative (**A**) control and (**B**) therapy animals. Tumors are marked with arrows; the color-bar represents the concentrations of the probe in nM.

**Figure 4:**
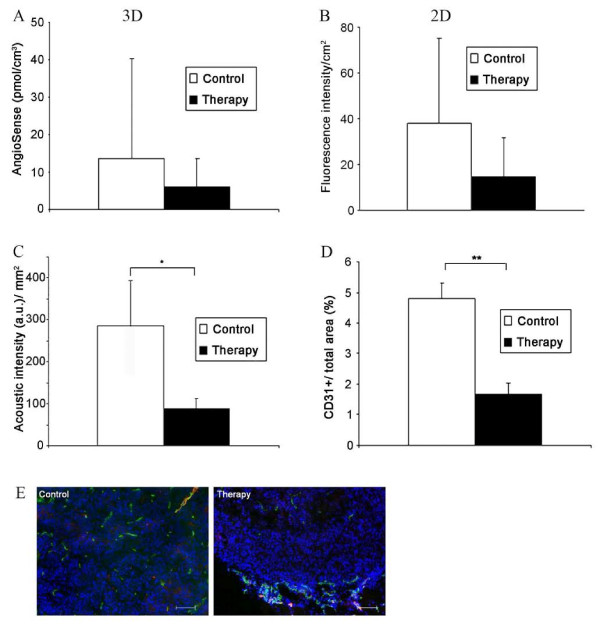
**Reduction in tumor vascularization after therapy**. (**A**) 3D data analysis reveals a higher mean concentration of AngioSense in the untreated controls (control, *n *= 7; therapy, *n *= 6), *P = *0.51. (**B**) 2D reflection data show an even stronger difference in fluorescence intensities between control and treated tumors, *P = *0.19. (**C**) Contrast-enhanced ultrasound shows a significantly higher acoustic intensity per tumor area in control versus treated tumors (*P < 0.01). (**D**) Quantification of the mean vessel density demonstrates a significantly higher vessel density in the control group, **P < 0.001. (**E**) Immunostaining of corresponding sections confirms the markedly increased vessel number in the controls; endothelial cells are stained with CD31 (green), smooth muscle cells with SMA (red), and cell nuclei with DAPI (blue), bar = 100 μm.

Higher AngioSense signals were detected by 3D optical imaging in the untreated control tumors (Figure 3A, B), and a higher mean AngioSense concentration was measured in the controls (Figure 4A; AngioSense/volume: control group, 13.8 ± 26.2 pmol/cm^3^; therapy group, 6.3 ± 7.3 pmol/cm^3^; *P *= 0.51). 2D reflection imaging even showed stronger differences in fluorescence intensity between the controls and treated tumors (Figure 4B; control group, 38 ± 37 FI/cm^2^; therapy group, 15 ± 17 FI/cm^2^; *P *= 0.19).

Contrast-enhanced ultrasound confirmed a significant decrease in tumor vascularization in treated tumors compared with untreated controls (Figure 4C; control, 285.3 ± 107.5 arbitrary units [a.u.]/mm^2^; therapy, 87.3 ± 23.9 a.u./mm^2^; *P < 0.01). The decreased vascularization in treated tumors was further validated by immunostaining of corresponding tumor sections and subsequent quantification of CD31-positive area fractions (control group, 4.8 ± 0.50%; therapy group, 1.7 ± 0.37%; **P < 0.001; Figure 4D; representative pictures are shown in Figure 4E). The restricted presence of SMA-positive vessels further indicated that in this model, the tumor vasculature is predominantly immature and therefore highly vulnerable to an antiangiogenic therapy (Figure 4E). In the treated tumors, SMA-positive, mature vessels were only detected at the tumor periphery (Figure 4E).

### Uptake of the radiotracer ^99 m^Tc-HYNIC-Annexin V in response to antiangiogenic treatment and analysis of vascularization

**Figure 5:**
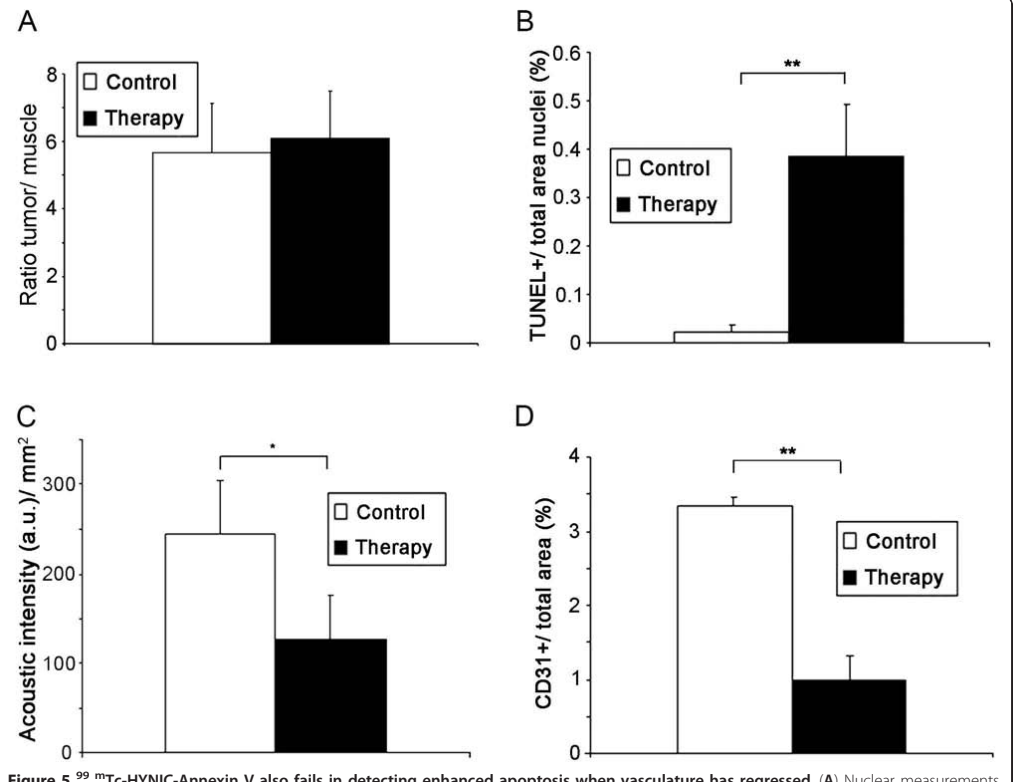
**^99 m^Tc-HYNIC-Annexin V also fails in detecting enhanced apoptosis when vasculature has regressed**. (**A**) Nuclear measurements using the tracer ^99 m^Tc-HYNIC-Annexin V reveal almost equal tumor-to-muscle ratios normalized to tissue weight in control and therapy tumors (control, *n *= 5; therapy, *n *= 6), *P = *0.63. (**B**) In contrast to nuclear measurements, quantification of the TUNEL-positive area fraction in sections of treated tumors demonstrates a highly significant increase in apoptosis in the treated tumors, **P < 0.001. (**C**) Contrast-enhanced ultrasound demonstrates again a significantly higher acoustic intensity per tumor area in control versus treated tumors (*P < 0.01). (**D**) Quantification of the mean vessel density in tumor sections demonstrates a highly significant decrease in vessel density in the therapy group, **P < 0.001.

In order to test whether the low accumulation of the NIRF-Annexin V probe in the treated tumors was due to the specificity problems of the probe or due to a reduced delivery at the target site (as a consequence of the therapy-induced vascular breakdown), a second therapy study was performed applying a similar therapy design. Instead of performing NIRF imaging, apoptosis was now quantified by radioactive measurements using the radiotracer ^99 m^Tc-HYNIC-Annexin V. Tumor vascularization was again determined in parallel by contrast-enhanced ultrasound. Two hours after application of the radiotracer ^99 m^Tc-HYNIC-Annexin V, the amount of radioactivity was measured *ex vivo *in the tumor, muscle, and liver by gamma counting. Interestingly, the tracer accumulation was similar in the tumors of the control group compared with the treated group, as shown by the almost equal tumor-to-muscle ratio normalized to the tissue weight (Figure 5A; control, 5.66 ± 1.46; therapy, 6.09 ± 1.40; *P *= 0.63). In contrast, immunohistology demonstrated again a significantly higher apoptosis rate in the treated tumors (Figure 5B; TUNEL-positive area: control, 0.023 ± 0.015%; therapy, 0.387 ± 0.105%; *P < 0.001).

In agreement with the first study, contrast-enhanced ultrasound revealed a significant decrease in the tumor vascularization in treated tumors compared with untreated controls (Figure 5C; control, 244.9 ± 58.4 a.u./mm^2^; therapy, 127 ± 50.1 a.u./mm^2^; *P < 0.01). This was in line with immunohistology, demonstrating a highly significant decrease in mean blood vessel density in tumors of the therapy group (Figure 5D; CD31-positive area: control group, 3.35 ± 0.112%; therapy group, 0.99 ± 0.324%; **P < 0.001).

## Discussion

Apoptosis imaging is discussed to have an important potential in the clinics for early disease detection, staging of disease progression, and the assessment of therapy effects [22]. In cardiovascular medicine, apoptosis imaging can improve the characterization of myocardial infarction, instable atherosclerotic plaques, and cardiac allograft rejection [10,23]. In addition, cancer therapies strongly induce apoptosis in the tumor tissue already at an early treatment stage and thus may be monitored effectively using apoptosis-specific molecular imaging probes [24]. ^99 m^Tc-Annexin V and ^99 m^Tc-HYNIC-Annexin V have shown to be promising tracers for assessing early effects of chemotherapy and radiation therapy in cancer patients [1,11,25].

In contrast to these findings, we report here that noninvasive apoptosis imaging as a sole technology is critical for therapy response evaluation, particularly when the therapy primarily affects the vasculature. This is predominantly the case for antiangiogenic therapies. Already, short-term treatment of SCC xenografts with the multi-tyrosine kinase inhibitor SU11248 for 4 days resulted in a strongly reduced vascularization of the tumor tissue, as indicated by optical imaging and clearly demonstrated by contrast-enhanced ultrasound and imunohistology. This observation is in line with previous studies about the effect of SU11248 on A431 tumor vessels [15,18]. In addition, immunohistology revealed strong pro-apoptotic effects of the antiangiogenic therapy on the tumor cells, especially within the almost devascularized center of the tumor, distant from mature and more functional blood vessels of the tumor periphery [15]. Contradictory to immunohistology, 2D optical reflection imaging and especially 3D fluorescence tomography showed strikingly lower concentrations of the NIRF probe Annexin Vivo in the treated tumors. These results pointed either to problems in probe delivery. On the other hand, since the data for both NIRF probes, Annexin Vivo and AngioSense, showed high standard deviations, we could not exclude problems in biodistribution. We therefore performed a second therapy study with a similar therapy design. Instead of performing NIRF annexin imaging, we used radiolabeled annexin (^99 m^Tc-HYNIC-Annexin V) and quantitatively determined the tracer uptake in the tumor by gamma counting. Almost equal tumor-to-muscle ratios normalized to tissue weight were obtained in therapy and control tumors, whereas immunohistology again demonstrated a highly significant increase in apoptosis in the treated tumors. This demonstrated that also nuclear medicine measurement of apoptosis using radiolabeled annexin failed in detecting increased apoptosis after antiangiogenic therapy. In agreement with the first study, contrast-enhanced ultrasound and immunohistology revealed a significantly reduced vascularization in SU11248-treated tumors. Thus, we conclude that the breakdown of the vasculature in response to the antiangiogenic treatment strongly impairs the delivery of the probe to the tumor tissue, consequently resulting in a low probe accumulation despite the enhanced apoptosis. This hypothesis is supported by the fact that the apoptotic tumor cells are predominantly located in the center with only a few remaining vessels and are distant from SMA-positive, mature blood vessels at the tumor periphery. The remaining functionality of SMA-positive, mature blood vessels in A431 tumors has been previously demonstrated by detecting blood flow within the mature vessels even after antiangiogenic treatment [15]. A431 tumors generally have a low amount of mature vessels as shown for the untreated controls, demonstrating the high vulnerability of the vasculature towards the antiangiogenic therapy. In treated tumors, the number of mature vessels was even lower than in the controls. Our conclusion of the problems in probe delivery due to antiangiogenic therapy is further supported by dynamic contrast-enhanced MRI of treated mouse colon xenografts, showing a reduced vascular permeability and vessel density as early as 24 h after antiangiogenic treatment [26,27]. Accumulation of probes or tracers in tissues with strongly reduced vascularization might be improved by using smaller molecules like short peptides instead of proteins since they would more easily penetrate and more easily be distributed in the tissue.

Thus, these results favor the use of annexin-based apoptotis imaging only for cancer therapies that have minor effects on the tumor vasculature like conventional chemotherapy or radiation. In addition, apoptosis imaging may be well suited for monitoring cell damage in disorders where the microvasculature is still present (e.g., reperfused ischemic areas post infarction or atherosclerotic vessels). However, for monitoring cancer therapy effects, our results recommend that apoptosis imaging should be supplemented by the analysis of tumor vascularization.

## Conclusions

The failure of annexin-based apoptosis assessment *in vivo *can be explained by the significant breakdown of the vasculature after therapy, resulting in reduced delivery of the probe or the tracer to the target tissue. Thus, annexin-based apoptosis imaging is only favorable for therapies that do not severely affect the vasculature.

## Abbreviations

au: arbitrary units; FI: fluorescence intensity; FMT: fluorescence molecular tomography; HYNIC: 6-hydrazinonicotinic; NIRF: near infrared fluorescence; PS: phosphatidylserine; ROI: region of interest; SCC: squamous cell carcinoma; SMA: alpha smooth muscle actin; TUNEL: terminal deoxynucleotidyl transferase-mediated dUTP nick end labeling.

## Competing interests

The authors declare that they have no competing interests.

## Authors' contributions

WL was mainly involved in the conception of the study, developed the methodology, performed experiments, analyzed the data, and wrote the manuscript. SA performed the experiments and analyzed the data. AR assisted in conducting the experiments and the analyses. FG performed the image reconstructions. DD assisted in the reconstructions and critically reviewed the manuscript. JSJ contributed to the nuclear measurements (synthesized the radioactive tracer) and critically reviewed the manuscript. FMM gave advice in the interpretation of the data and critically reviewed the manuscript. FK provided advice in the conception of the study, interpretation of the data, and critically reviewed the manuscript. MP provided important advice in the conception of the study, performed experiments, assisted in data analysis and interpretation, and critically reviewed the manuscript. All authors approved the final manuscript.
